# Histone acetyltransferases and deacetylase 6 (HDAC6) Promotes Hepatic Stellate Cell Activation and Liver Fibrosis

**DOI:** 10.7150/ijbs.137447

**Published:** 2026-09-10

**Authors:** Ji Hyun Lee, Ann-Yae Na, Ji Hye Kim, Tae Hyun Park, Ji Hye Yang, Joo Yong Lee, Chang Yeob Han, Sohi Kang, IL Je Cho, Sangkyu Lee, Sung Hwan Ki

**Affiliations:** 1 MRC-OSTRC, Research Institute of Pharmaceutical Sciences, College of Pharmacy, Chosun University, Gwangju, 61452, Republic of Korea.; 2 Honam Regional Future Drug Development Convergence Education & Research Division (BK21 FOUR), Chosun University, Republic of Korea.; 3 KNU G-LAMP Project Group, KNU Institute of Basic Sciences, Kyungpook National University, Daegu 41566, Republic of Korea.; 4 College of Korean Medicine, Dongshin University, Naju, Jeollanam-do, 58245, South Korea.; 5 Graduate School of Analytical Science and Technology, Chungnam National University, Daejeon, Korea.; 6 School of Pharmacy, Jeonbuk National University, Jeonju, 54896, Republic of Korea.; 7 Department of Anatomy and Convergence Medical Science, College of Medicine, Institute of Health Sciences, Gyeongsang National University, Jinju 52727, Republic of Korea.; 8 Central Research Center, Okchundang Inc., Daegu 41059, Republic of Korea.; 9 School of Pharmacy, Sungkyunkwan University, Suwon 16419, Republic of Korea.

**Keywords:** liver fibrosis, hepatic stellate cell, proteomics, HDAC6, ACAT1

## Abstract

Liver fibrosis results from excessive extracellular matrix (ECM) deposition following chronic hepatocyte injury. Hepatic stellate cell (HSC) activation is central to fibrogenesis; however, the regulatory mechanisms controlling this process remain unclear. Protein acetylation, regulated by histone acetyltransferases and deacetylases (HDACs), modulates gene expression and protein stability. To date, no studies have explored acetylation dynamics during HSC activation using proteomic approaches. In this study, we investigated the role of acetylation in HSC activation and liver fibrosis. Proteomic profiling of primary mouse HSCs was performed to assess global protein acetylation and the expression of HDACs and SIRTs during HSC activation. Activated HSCs exhibited reduced global protein acetylation, increased HDAC2/3/6 expression, and decreased SIRTs expression. Selective inhibition of HDAC6, but not HDAC2/3, suppressed TGF-β-induced HSC activation. TGF-β increased HDAC6 expression via Smad3-dependent transcription, and HDAC6 promoted fibrogenic gene expression through Smad activation. Proteomic screening identified acetyl-CoA acetyltransferase 1 (ACAT1) as a novel HDAC6 substrate; HDAC6 overexpression enhanced ACAT1 binding and reduced its acetylation. *In vivo*, pharmacological inhibition of HDAC6 lowered ALT and AST levels and improved histological changes in fibrotic mice. Our results suggest that selective HDAC6 inhibition may represent a promising therapeutic strategy for treating liver fibrosis.

## Introduction

Liver fibrosis is a pathological response to chronic inflammation, characterized by extensive hepatocyte necrosis and the deposition of extracellular matrix (ECM) within healthy liver tissue 1. Excessive ECM accumulation disrupts the structure and function of the liver, potentially leading to irreversible fibrosis and cirrhosis, which are substantial contributors to chronic liver diseases 2, 3. However, current treatment options for liver fibrosis are limited, highlighting the urgent need to develop new therapies. Recent studies have implicated epigenetic regulation as a key factor in fibrogenesis, providing valuable insights into the development of new therapies for chronic liver diseases, although current results remain controversial 4-7.

Epigenetic regulation has been studied in the activation of hepatic stellate cells (HSCs), which play a major role in liver fibrosis. However, few studies have explored the regulatory mechanism of acetylation. Histones are a family of basic proteins rich in lysine and arginine residues. They have a globular domain as well as *N*- and *C*-terminal tails that undergo various post-transcriptional modifications, the most well-characterized of which are methylation, phosphorylation, ubiquitylation, and acetylation 8, 9. Histone acetylation is a dynamic and reversible process involving the addition of an acetyl group to lysine residues 10. This modification is generally associated with transcriptional activation and regulated by two sets of enzymes: histone acetyltransferases (HATs) and histone deacetylases (HDACs). In mammals, 18 HDACs have been identified and are categorized into two main types and four subclasses based on their structure and mechanism of action: Classes I (HDAC 1, 2, 3, and 8), II (HDAC 4, 5, 6, 7, 9, and 10), III (SIRT 1-7); and IV (HDAC 11). Classes I, II, and IV HDACs are Zn^2+^-dependent, whereas class III HDACs are nicotinamide adenine dinucleotide (NAD^+^)-dependent 11, 12. Several studies have highlighted complex alterations in HDAC activity during liver fibrosis 13. However, conflicting views regarding the pathogenic role of HDAC-mediated deacetylation have hindered the development of novel therapeutic strategies targeting HDACs in liver fibrosis.

Among the 18 HDACs identified in humans, HDAC6 is unique because it contains two tandem catalytic domains (CD1 and CD2) located in the *N*-terminal and central regions, respectively, and a zinc finger ubiquitin-binding domain located in the *C*-terminal region. It preferentially localizes in the cytoplasm 14. HDAC6 plays key roles in microtubule stability, the maintenance of cellular function, inflammation, cell survival, and proliferation 15, 16. Furthermore, HDAC6 is a critical biological regulator involved in the degradation of misfolded proteins and modulation of various cellular signaling pathways through the deacetylation of non-histone substrates and interactions with other proteins 17, 18. Notably, HDAC6 has emerged as a pivotal molecule mediating the development and progression of liver cancer, making it a prominent target in anticancer drug research 19. HDAC6 has primarily been studied in liver cancer; however, changes in protein acetylation and the regulation of specific HDAC isoforms during HSC activation remain unclear, and the underlying regulatory mechanisms require further investigation.

In this study, we explored which HDAC isoforms contribute to liver fibrosis and their regulatory mechanisms. We identified that HDAC6 plays a role in the decreased acetylation observed during HSC activation and investigated its regulatory role in this process. Our analysis revealed that acetyl-CoA acetyltransferase 1 (ACAT1) is a novel substrate of HDAC6. Furthermore, we evaluated the effects of pharmacological inhibition of HDAC6 in animal models of liver fibrosis. Collectively, our findings highlight the role of HDAC6 as a promising therapeutic target for the prevention and treatment of liver fibrosis.

## Results

### Decreased acetylation during HSC activation is associated with HDAC6 upregulation

Activation of HSCs is the main source of ECM production during liver fibrosis. To observe significant changes in acetylation at the protein level between HSC days 0 and 7, we performed western blot analysis of quiescent and activated primary HSCs. Global protein acetylation decreased in activated HSCs (Figure 1A). These results suggest that HSC activation may regulate changes in protein acetylation. Acetylation is regulated by the opposing activities of HATs and HDACs. Therefore, we hypothesized that HSC activation may influence acetylation changes through the regulation of HDACs.

To examine the relationship between HDACs and HSC activation, we systematically measured the expression of HDAC1-7 (except HDAC4) and SIRT1-7 in primary HSCs. The levels of HDAC2, HDAC3, and HDAC6 were markedly increased in activated HSCs, whereas those of the other HDACs were decreased (Figure 1B). In contrast, the expression of SIRTs was not increased by HSC activation but decreased. Significantly higher levels of HDAC2, HDAC3, and HDAC6 were observed in the CCl_4_-induced liver fibrosis mouse model (Figure 1C). TGF-β1 is a major fibrogenic factor that promotes HSC activation. Subsequently, we investigated which of the increased HDACs could modulate TGF-β-induced fibrogenic gene expression. Treatment with trichostatin A, a pan-HDAC inhibitor, significantly inhibited TGF-β-induced plasminogen activator inhibitor-1 (PAI-1), which is an acute phase protein known to correlate with hepatic fibrosis (Figure 1D). However, santacruzamate A, an HDAC2 selective inhibitor, and RGFP966, an HDAC3 inhibitor, failed to affect TGF-β-mediated fibrogenic gene expression (Figure 1E). In contrast, two selective HDAC6 inhibitors, tubastatin A (TST) and ACY-1215, markedly attenuated TGF-β1-induced PAI-1 expression (Figure 1F). Collectively, these findings indicate that HDAC6 could decrease acetylation during HSC activation and regulate TGF-β signaling in liver fibrosis.

### HDAC6 is induced during HSC activation

Acetylation markedly decreased upon HSC activation, a process specifically regulated by HDAC6. Analysis of GEO datasets revealed elevated HDAC6 expression in liver samples from patients with fibrosis and in activated HSCs (Figures 2A and 2B). HDAC6 expression was also detected in liver biopsies of patients with liver cancer. HDAC6 protein levels increased in cirrhotic livers (Figure S1). In addition, TGF-β-induced upregulation of HDAC6 was confirmed in primary mouse HSCs (Figure 2C). We further examined the time-dependent induction of HDAC6 following TGF-β treatment and found that HDAC6 levels increased within 1-12 h (Figure 2D). Dose-response analysis showed that HDAC6 expression was upregulated by TGF-β stimulation at concentrations up to 2 ng/mL (Figure 2E). Consistently, TGF-β treatment enhanced HDAC6 immunostaining intensity (Figure 2F). These findings highlight the robust induction of HDAC6 expression in activated HSCs during liver fibrogenesis.

### TGF-β-mediated HDAC6 induction occurs via a Smad3-dependent pathway

To investigate the regulatory mechanism of HDAC6 induction, we measured HDAC6 mRNA levels and found that they were elevated following TGF-β treatment, consistent with the changes observed at the protein levels (Figure 3A). When cells were treated with TGF-β in the presence of actinomycin D, a transcriptional inhibitor, the TGF-β-induced increase in HDAC6 expression was abolished (Figure 3B). Since TGF-β signaling is primarily mediated by the canonical Smad pathway, and activated Smad proteins regulate cellular functions by modulating target gene expression, we subsequently examined the role of Smad3 on TGF-β-induced HDAC6 expression. Treatment with SIS3, a specific Smad3 inhibitor, markedly suppressed TGF-β-induced HDAC6 upregulation (Figure 3C). Conversely, TGF-β treatment enhanced HDAC6 expression in mock-transfected cells, and this effect was further enhanced by Smad3 overexpression (Figure 3D). To assess whether Smad3 directly activates HDAC6 transcription, we constructed a plasmid containing the human HDAC6 promoter (pGL415-phHDAC6) and performed luciferase reporter assays. HDAC6 promoter luciferase activity was significantly increased by either Smad3 overexpression or TGF-β treatment (Figure 3E). These results demonstrate that TGF-β-mediated HDAC6 induction occurs via a Smad3-dependent transcriptional mechanism.

### HDAC6 promotes liver fibrogenesis

To investigate whether HDAC6 affects liver fibrogenesis, we examined the effects of HDAC6 overexpression on PAI-1 expression. PAI-1 levels were significantly increased by HDAC6 transfection following TGF-β stimulation (Figure 4A). The Smad3-dependent regulatory roles of TGF-β signaling in liver fibrosis are well documented. To further investigate the mechanism by which HDAC6 drives the fibrotic response, we explored the role of Smad3 signaling in TGF-β-mediated liver fibrogenesis. Treatment of mock-transfected cells with TGF-β resulted in increased Smad3 phosphorylation, and HDAC6 overexpression further enhanced TGF-β-induced Smad3 phosphorylation (Figure 4B). HDAC6 deacetylates tubulin, a microtubule subunit, and cortactin, an actin-associated protein, thereby influencing cytoskeletal dynamics. HDAC6 promotes the migration and invasion of liver cancer cells by modulating these dynamics 20. This prompted us to perform wound healing assay to examine the effect of HDAC6 on TGF-β-mediated migration. Treatment with TGF-β significantly increased cell migration; however, migration into the wound area was markedly reduced after treatment with the TST (Figure 4C). Moreover, TST treatment attenuated TGF-β-mediated Smad3 phosphorylation and inhibited SBE luciferase activities (Figures 4D and 4E). These results suggest that HDAC6 overexpression in HSCs facilitates the activation of Smad3, thereby promoting fibrogenesis.

### Landscape of protein lysine acetylation in HSCs

To characterize the reduced protein lysine acetylation (Kac) associated with HSC activation, a global acetylome study was performed by comparing HSCs on days 0 and 7. To assess changes in acetylated proteins and identify their Kac sites in the proteome between the day 0 and 7 HSC groups, we incorporated H_2_^16^O (light, L) labelling for the day 0 group and H_2_^18^O (heavy, H) labelling for the day 7 group in a quantitative proteomic approach (Figure 5A). ^16/18^O-labeled peptides were combined; 95% of the mixed peptides were enriched by immunoaffinity purification of acetylated peptides using anti-acetyl-lysine-conjugated agarose beads, and the remaining 5% were analyzed by LC-MS/MS after high-pH peptide fractionation.

In this study, we identified 613 non-redundant monoacetylated peptides corresponding to 679 Kac sites on 337 Kac proteins. Notably, most Kac proteins exhibited decreased abundance at day 7 (H) compared with day 0 (L) in HSCs, consistent with the reduced Kac levels observed at day 7 by immunoblot analysis using a pan-antibody (Figure 1A; Figure 5B). Of the 679 identified Kac sites in the Venn diagram, 601 (88.5%) were present in the day 0 group, six sites (0.9%) in the day 7 group, and 72 sites (10.6%) in both groups. Of the 337 Kac proteins, 323 were observed only at day 0, six Kac protein observed in both groups, and only eight Kac proteins were observed at day 7.

To characterize Kac across the proteome, 337 Kac proteins were analyzed for GO terms and KEGG pathways using the DAVID database. Figures 5C‒E show Gene Ontology (GO) enrichment analysis of Kac-containing proteins, with a pie chart representing -log10 (Fisher's exact test). Among the 14 upregulated Kac proteins (fold change [FC] ≥ 1.5 and detected only in the day 7 group), GO molecular function (GOMF) analysis showed that these proteins predominantly belonged to the structural constituent of chromatin category, with protein heterodimerization activity representing the second-largest category (Figure 5C). Analysis of the GO Biological Process (GOBP) domain suggested that Kac proteins are mainly involved in the negative regulation of megakaryocyte differentiation, DNA replication-dependent nucleosome assembly, and DNA replication-independent nucleosome assembly. In the GO Cellular component (GOCC) category, upregulated Kac proteins were enriched in CENP-A-containing nucleosomes, nuclear chromosomes, and nucleosomes. For the 329 downregulated Kac proteins (FC ≤ 0.66 and detected only in the day 0 group), GOCC analysis revealed enrichment in mitochondria and the mitochondrial inner membrane, whereas GOBP analysis indicated involvement in mitochondrial ATP synthesis coupled to proton transport and negative regulation of megakaryocyte differentiation (Figure 5D). Furthermore, the GOMF domain of the downregulated Kac proteins was enriched in oxidoreductase activity and structural constituents of chromatin. KEGG pathway analysis demonstrated significant over-representation of the metabolic pathway, diabetic cardiomyopathy, chemical carcinogenesis, oxidative phosphorylation, and Huntington's disease (Figure 5E).

### Investigation of the Kac substrate of HDAC6

Many proteins execute their cellular functions through interactions with binding partners, and the loss of specific protein-protein interactions (PPIs) in organisms can lead to various diseases. Post-translational modifications can serve as docking sites for recruiting binding proteins and may regulate PPIs. Using the STRING database v12.0, we constructed PPI networks of downregulated Kac proteins to elucidate the molecular mechanisms underlying liver fibrosis following HSC activation. After removing disconnected nodes, a highly connected protein interaction network (0.9) was obtained, and the data were imported for visualization and further network analysis (Figure S2). To explore the role of ACAT1 in the regulation of PPIs through Kac, we constructed protein interaction networks with ACAT1-regulated Kac proteins (Figure 5F). The interaction network highlighted that ACAT1 was highly interconnected, especially in cluster 1, which was associated with valine, leucine, and isoleucine degradation; metabolic pathways; propanoate pathways; and fatty acid degradation based on KEGG pathway analysis (Figure 5G). In this study, seven Kac sites were identified on the ACAT1 protein, with four Kac sites quantified exclusively in the HSC day 0 group (Table S1).

Subsequently, we identified potential HDAC6 substrates and clarified their underlying mechanisms. HDACs regulate gene expression by inducing closed chromosomal conformation. Therefore, we also examined global proteomic changes (Figure 6). Of the 4825 identified proteins in the Venn diagram, 2964 (61.4%) were present in the day 0 group, seven (0.1%) in the day 7 group, and 1854 (38,4%) in both groups (Figure 6A). The 446 DEPs identified in the global proteomics were analyzed for GO terms and KEGG pathways using the DAVID database. Figures 6B and 6C show GO enrichment analysis of these proteins, with a pie chart representing -log10 (Fisher's exact test). Among the 21 upregulated proteins (FC ≥ 1.5), GOMF analysis indicated enrichment in the protein binding category. Analysis of the GOBP domain suggested involvement in the activation of cysteine-type endopeptidase activity and actin filament bundle assembly. Regarding GOCC, the upregulated proteins were enriched in actin filaments and cytoplasm. For the 425 downregulated proteins (FC ≤ 0.66), GOCC analysis revealed enrichment in extracellular exosomes, mitochondria, and the mitochondrial inner membrane. The GOMF domains of the down-regulated proteins were enriched in oxidoreductase activity, electron carrier activity, and poly(A) RNA binding. Furthermore, GOBP analysis indicated involvement in oxidation-reduction processes, transport, and metabolic processes. KEGG pathway analysis showed significant enrichment in metabolic pathways; oxidative phosphorylation; and valine, leucine, and isoleucine degradation. As shown in Figure 6, ACAT1, which is highly interconnected, was identified as a key down-regulated DEP. Consistent with the GO and KEGG analyses of downregulated DEPs, ACAT1 was enriched in metabolic processes in GOBP, mitochondria, and inner mitochondrial membrane in GOMF, and associated with metabolic pathways, carbon metabolism, and fatty acid metabolism in KEGG analysis.

### ACAT1 is a novel substrate of HDAC6

ACAT1 is an enzyme that catalyzes the reversible formation of acetoacetyl-CoA from two molecules of acetyl-CoA and is involved in carcinogenesis and fibrogenesis 21, 22. Using a proteomic approach, ACAT1 was identified as an unreported HDAC6 binding partner. To determine whether fibrogenic signaling regulates ACAT1 expression in HSCs, we initially analyzed ACAT1 expression during HSC activation in primary cultured murine HSCs. ACAT1 was abundant in quiescent HSCs, as detected by immunoblotting, and its expression decreased in activated HSCs (Figure 7A). Treatment of LX-2 cells with TGF-β also reduced ACAT1 expression (Figures 7B and 7C). Subsequently, we ectopically expressed HDAC6 in cells to confirm that ACAT1 is a downstream target of HDAC6. Immunoprecipitation experiments demonstrated the formation of HDAC6-ACAT1 complexes (Figure 7D), suggesting that HDAC6 may deacetylate ACAT1. Analysis of the acetylation levels of HDAC6 revealed that, although the general ACAT1 levels remained unchanged, ACAT1 acetylation decreased by HDAC6 overexpression (Figure 7E). These data suggest that ACAT1 is a specific downstream target of HDAC6 in HSCs. Finally, we assessed whether ACAT1 regulates hepatic fibrogenesis. PAI-1 levels after treatment with TGF-β were decreased by ACAT1 (Figure 7F). These results suggest that ACAT1 may be associated with HSC activation.

### TST suppresses liver fibrosis *in vivo*

Subsequently, we investigated whether the *in vitro* findings could be replicated *in vivo*. TST has therapeutic effects in several diseases 17, 23, 24. TST treatment inhibited the increase in serum ALT levels, whereas AST levels were not significantly changed (Figure 8A). In H&E-stained liver tissues, pathological changes were observed in CCl_4_-injected mice. However, TST treatment attenuated inflammatory cell infiltration, focal hepatocellular necrosis, and CCl_4_-induced hepatocyte ballooning (Figure 8B). In contrast, TST-treated mice significantly inhibited CCl_4_-induced collagen accumulation (Figure 8C). In addition, the levels of fibrosis markers elevated by CCl_4_ treatment were inhibited by TST (Figure 8D). Consistently, mRNA levels of α-SMA in the liver homogenates of the CCl_4_-induced fibrosis animal model were reversed by TST treatment (Figure 8E). These findings suggest that HDAC6 inhibition by TST protects against CCl_4_-induced liver fibrosis.

## Discussion

HSC activation plays a central role in liver fibrosis development 27. In healthy livers, HSCs remain quiescent and participate in lipid metabolism. However, repeated stimulation triggers their activation and transformation into ECM-producing cells, driving the progression of fibrosis 28. Current research has focused on modulating signaling pathways in HSCs to control fibrosis onset and progression 29, 30. Despite these efforts, studies on the key molecules and mechanisms that regulate HSC activation and fibrosis remain limited, partly because most studies have targeted specific gene expression changes or pathways without addressing broader regulatory networks 31, 32. Therefore, establishing new paradigms for the prevention and treatment of fibrosis remains essential.

In this study, we performed a proteomic analysis of protein acetylation changes during HSC activation and identified HDAC6 as a potential target for fibrogenesis. Notably, no previous studies have used a proteomic approach to identify targets that control HSC activation through acetylation changes. Proteomic profiling of primary murine HSCs revealed a significant decrease in acetylated proteins during HSC activation (Figure 5), which was confirmed by acetyl-lysine antibody experiments (Figure 1A). We hypothesized that the reduction in protein acetylation during HSC activation is driven by HDAC- or SIRT-mediated deacetylation. By evaluating HDAC and SIRT expression during HSC activation, we observed increased expression of HDAC2, HDAC3, and HDAC6; however, the expression of other HDACs or SIRTs did not increase. In particular, SIRT expression was decreased in activated HSCs compared with quiescent HSCs. Further studies are required to determine the role of decreased SIRT expression in HSC activation. To identify the specific HDAC isoforms involved, we tested selective inhibitors of HDAC2, HDAC3, and HDAC6 on TGF-β-induced HSC activation. Santacruzamate is a highly potent HDAC2 inhibitor with an IC_50_ of 0.112 nM 33, whereas RGFP966 selectively inhibits HDAC3 with an IC_50_ of 0.08 nM and minimal effect on other HDAC isoforms 34. TST has an IC_50_ value of 15 nM and demonstrates over 1,000-fold selectivity against all isoforms except HDAC8 (57-fold selectivity) 35. Another HDAC6 inhibitor, ACY-1215, exhibits stronger inhibitory activity toward HDAC6 than the other isoforms 36. Among these inhibitors, HDAC6 inhibitors effectively suppressed TGF-β-mediated HSC activation, identifying HDAC6 as a key regulator of HSC activation. HDAC enzymes influence various diseases by modulating cell signaling and metabolism 37, 38 and have emerging roles in metabolic and liver diseases 6, 7, 39. Previous studies have demonstrated that HDAC inhibitors improve fatty liver disease and regulate HSC activation 40-42; however, the mechanisms linking protein acetylation changes and HDAC expression during HSC activation remain unclear.

TGF-β signaling, crucial in chronic liver fibrosis, primarily activates Smad2/3, which form complexes with Smad4 to induce profibrogenic genes, while also triggering Smad-independent pathways such as AP-1 43-45. TGF-β induced HDAC6 expression in LX-2 and HSC-T6 HSC cell lines (Figure 2 and Figure S3). HDAC6 induction is transcriptionally regulated via the Smad3 signaling pathway: HDAC6 mRNA increased after TGF-β treatment and was sensitive to actinomycin-D, a transcription inhibitor. Smad3-specific inhibition or Smad3 overexpression experiments confirmed Smad3-dependent regulation of HDAC6 expression. The non-canonical TGF-β pathway via AP-1 was not associated with HDAC6 induction (Figure S4). However, the direct Smad3 binding site in the HDAC6 promoter remains unknown. Functionally, TGF-β-induced HDAC6 promoted HSC activation, enhancing the expression of the fibrogenic marker PAI-1 (Figure 4). HDAC6 overexpression increased PAI-1, whereas selective HDAC6 inhibitors reduced TGF-β-induced HSC activation and cell migration (Figure 4C and Figure S5A). Furthermore, HDAC6 overexpression amplified both Smad and AP-1 activities, creating a positive feedback loop that enhanced fibrogenesis in HSCs (Figure 4E and Figure S5B).

Proteomic analysis identified ACAT1 as a novel HDAC6 substrate (Figures 5 and Figure S2). ACAT1 directly interacts with HDAC6 and is deacetylated by HDAC6 (Figures 7D and 7E). Seven acetylation sites on ACAT1 were identified (Table S1), and their roles as HDAC6 substrates require further investigation. ACAT1, a mitochondrial enzyme that catalyzes the reversible formation of acetoacetyl-CoA from acetyl-CoA, participates in fatty acid oxidation, ketogenesis, and other metabolic pathways 22. We focused on ketone metabolism owing to its emerging role in liver diseases. Ketone bodies, such as acetoacetate and β-hydroxybutyrate, serve as energy sources during glucose scarcity 46. Ketogenic diets can prevent fatty liver by enhancing hepatic fat oxidation 47, and ketone metabolism is reduced in patients with metabolic-associated steatohepatitis 48. Our findings suggest that the modulation of ketone metabolism via HDAC6-ACAT1 may offer a novel strategy to prevent or treat liver fibrosis.

To explore the *in vivo* role of HDAC6, we tested whether HDAC6 inhibitors improve liver fibrosis induced by CCl_4_ treatment. Treatment with TST significantly attenuated CCl_4_-induced liver injury and fibrosis (Figure 8). TST reduced serum ALT/AST levels, hepatocyte necrosis, edema, inflammatory infiltration, and expression of α-SMA and PAI-1. These results suggest that HDAC6 inhibitors could effectively attenuate liver fibrosis.

In summary, TGF-β/Smad3 signaling upregulates HDAC6 in HSCs, promoting HSC activation and liver fibrogenesis. Moreover, HDAC6 inhibitor attenuate these effects both *in vitro* and *in vivo*. ACAT1 is a newly identified HDAC6 substrate involved in ketone metabolism, revealing the HDAC6-ACAT1 axis as a promising therapeutic target for liver fibrosis.

## Methods

### Materials

Phospho-Smad3, Smad2/3, acetylated lysine, HDAC6, SIRT3, SIRT6, and SIRT7 antibodies were obtained from Cell Signaling Technology (Danvers, MA, USA). SIRT2, SIRT4, SIRT5, HDAC1, HDAC2, HDAC3, HDAC5, HDAC6, and HDAC7 antibodies were purchased from Abcam (Cambridge, UK). SIRT1 antibody was obtained from NOVUS Biologicals (Littleton, CO, USA). Antibodies against GAPDH and acetylated α-tubulin were acquired from Ab Frontier (Seoul, Korea) and Santa Cruz Biotechnology (Santa Cruz, CA, USA), respectively. PAI-1 antibody was purchased from BD Biosciences (Franklin Lakes, NJ, USA), whereas the ACAT1 antibody was sourced from Proteintech (Rosemont, IL, USA). β-actin and α-SMA antibodies, along with actinomycin D, trichostatin A, and SIS3, were obtained from Sigma (St. Louis, MO, USA). TGF-β was provided by R&D Systems (Minneapolis, MN, USA). HRP-conjugated goat anti-mouse and anti-rabbit antibodies were purchased from Invitrogen (Carlsbad, CA, USA). Santacruzamate A, RGFP966, tubastatin A (TST), and ACY-1215 were purchased from Selleckchem (Houston, TX, USA). The TGF-β antagonist SB431542 was acquired from Cayman Chemical (Ann Arbor, MI, USA).

### Cell culture

The human HSC line LX-2 was provided by Dr. S.L. Friedmann (Mount Sinai School of Medicine, NY, USA), and the rat HSC line HSC-T6 was obtained from Merck Millipore (Burlington, MA, USA). Both cell lines were cultured in DMEM supplemented with 10% fetal bovine serum (Atlas Biologicals, Fort Collins, CO, USA), 50 U/mL penicillin, and 50 µg/mL streptomycin, under a humidified atmosphere containing 5% CO₂ at 37°C.

### Isolation of HSCs

HSCs were isolated from mice using a previously established protocol 49. Briefly, following portal vein cannulation, the liver was perfused in situ with calcium-free HBSS at 37°C for 15 min, followed by perfusion with a solution containing 0.05% collagenase and calcium for 20 min at a flow rate of 10 mL/min. The perfused liver was then dissected, passed through a 70 µm cell strainer (BD Biosciences), and centrifuged at 50 × g for 2 min to separate parenchymal from nonparenchymal cells. The non-parenchymal cell fraction was further processed by centrifugation at 500 g for 10 min, resuspended in a Ficoll-Percoll gradient (1:9; GE Healthcare, IL, USA), and centrifuged at 1,400 g for 20 min. HSCs were collected from the interphase. Quiescent HSCs were cultured for 0 days, whereas activated HSCs were cultured for 7 days.

### Western blotting

SDS-PAGE and immunoblotting were performed as previously described 50. Cells were lysed in RIPA buffer for 1 h, and the resulting lysates were centrifuged at 12,000 × g for 10 min to extract proteins. Protein samples were separated by SDS-PAGE, transferred to nitrocellulose membranes, and probed with specific antibodies. Protein signals were detected using chemiluminescent substrates (Thermo Fisher Scientific).

### RT-PCR analysis

Total RNA was extracted from cells or tissues using TRIzol reagent (Invitrogen) based on the instructions of the manufacturer and reverse transcribed into cDNA. cDNA was synthesized using AccuPower® RT PreMix (Bioneer, Daejeon, Korea) in a thermal cycler (Bio-Rad, Hercules, CA, USA). PCR-amplified products were analyzed on an agarose gel, stained with ethidium bromide (Sigma-Aldrich), and visualized using a gel documentation system (Fujifilm, Tokyo, Japan). The primer sequences used were as follows: HDAC6 (Human) F 5'-GCCTCAATCACTGAGACCATCC-3', and R 5'-GGTGCCTTCTTGGTGACCAACT-3'; GAPDH (Human) F 5'-GAAGGTGAAGGTCGGAGTC-3', and R 5'-GAAGATGGTGATGGGATTTC-3'; a-SMA (Mouse) F 5'-TCCTCCCTGGAGAAGAGCTAC-3' and R 5'-TATAGGTGGTTTCGTGGATGC-3'; GAPDH (Mouse) F 5'-TGCCCCCATGTTTGTGATG-3' and R 5'-TGTGCTCATGAGCCCTTCC-3'.

### Gene Expression Omnibus (GEO) database

Gene expression profiles associated with liver fibrosis were obtained from the public repository of the NCBI GEO under accession numbers GSE68000 and GSE139602.

### Transient transfection experiment

HDAC6-FLAG plasmid was obtained from Addgene (Watertown, MA, USA). The ACAT1 (Myc-DDK-tagged) plasmid was purchased from ORIGENE (Rockville, Maryland, USA). Transient transfection was conducted using plasmids with LipofectamineTM2000 reagent (Invitrogen) based on the guidelines of the manufacturer.

### Wound healing assay

Wound healing assay was performed as previously described 50. Cells were cultured in six-well plates, and the culture inserts were removed when the cell density reached 90%. Subsequently, the cells were washed and treated with TST in the presence or absence of TGF-β. Samples were observed under a microscope (Axiovert 200 m; Carl ZEISS, Baden-Württemberg, Germany).

### Immunofluorescence staining

Cells were fixed with 4% paraformaldehyde for 20 min at room temperature (RT) and permeabilized with 0.1% Triton X-100 in 1X PBS for 10 min. Following permeabilization, the cells were incubated with an HDAC6 antibody overnight at 4°C, followed by incubation with a secondary antibody for 1 h at RT. Samples were mounted onto slides using a mounting solution. Stained samples were analyzed using the EVOS™ M5000 Cell Imaging System (Invitrogen).

### Construction of plasmid

Genomic DNA from HepG2 was used to amplify the human HDAC6 promoter region from -1994 to +12 bp using specific primers containing KpnI or XhoI recognition sites. The amplified fragment was inserted into the pGL4.15 plasmid (Promega, Madison, WI, USA) to generate pGL415-phHDAC6. Transfection was performed using LipofectamineTM2000. Firefly and Renilla luciferase activities were measured using the Dual-Luciferase Assay System (Promega), and relative luciferase activity levels were calculated.

### Luciferase reporter assay

Smad-binding element (SBE)-Luc or AP-1-Luc cells were transfected with the pRL-TK plasmid using LipofectamineTM2000 reagent (Invitrogen). Subsequently, transfected cells were treated with TST in the presence or absence of TGF-β for another 12 h. Firefly and Renilla luciferase activities of the cell lysates were measured using the Dual-Luciferase Assay System (Promega, Madison, WI, USA) based on the instructions of the manufacturer.

### Patient samples

The study was conducted in accordance with the Declaration of Helsinki, and the protocol was approved by the Ethics Committee of Jeonbuk National University Hospital in Korea (IRB number: CUH 2023-11-024-001).

### Animals

All animal experiments were conducted with the approval of the Animal Care and Use Committee of Chosun University and in accordance with the institutional ethical guidelines. ICR mice (6 weeks old) were procured from Oriental Bio (Sungnam, Korea) and acclimatized for 1 week before experimentation. Mice (n = 8 per group) were housed at 25 ± 2°C under a 12-h light/dark cycle with relative humidity maintained at 50 ± 5%, in pathogen-free conditions. Food (Oriental Bio) and water were provided *ad libitum*.

### CCl_4_-induced hepatic fibrosis

To induce liver fibrosis, ICR mice were administered carbon tetrachloride (CCl_4_) in olive oil (0.5 mL/kg) via intraperitoneal injection three times per week for 2 weeks. For the treatment group, TST (25 or 50 mg/kg; dissolved in 5% DMSO, 40% PEG400, 5% Tween, and 50% distilled water) was administered orally five times per week for the same duration. Mice were sacrificed 24 h after the final injection, and blood and tissue samples were collected for further analysis.

### Blood biochemistry

Serum alanine aminotransferase (ALT) and aspartate aminotransferase (AST) levels were measured using ALT/AST assay kits (Asan Pharmaceutical, Seoul, Korea).

### Histopathology

Liver tissue samples were trimmed along the sagittal axis and fixed in 10% neutral-buffered formalin for 24 h. After fixation, tissues were processed using an automated tissue processor and embedding system. Paraffin blocks were sectioned into 3-4 μm slices, with three serial sections prepared per block using a microtome. Representative sections were stained with hematoxylin and eosin (H&E) for general histopathology and with Sirius Red for collagen fiber detection. Degenerative hepatocytes, characterized by necrosis, eosinophilic condensation, and ballooning, were identified using H&E and Sirius Red staining. Hepatic architectural changes and fibrosis were evaluated using a modified hepatic staging scoring system. All analyses were conducted by a certified histopathologist blinded to sample identities.

### Sample preparations for comparative proteome analysis

Cell pellets from the HSC 0-day (n=3) and 7-day (n=3) groups were lysed by sonication in RIPA buffer containing Halt protease inhibitor cocktail, 1x phosphatase inhibitor, nicotinamide, and sodium butyrate. Lysates were centrifuged at 12,000 × g for 10 min at 4°C to remove cell debris. The supernatant was transferred to a new tube, and the same amount of protein from each group was prepared using a bicinchoninic acid colorimetric assay kit (Thermo Fisher Scientific). For reduction and alkylation, samples were treated with 15 mM dithiothreitol at 56°C for 45 min, followed by alkylation with 60 mM iodoacetamide for 30 min at RT in the dark. Proteins were purified by adding 10% trichloroacetic acid (TCA) and incubating at -4°C for 4 h, followed by two washes with ice-cold acetone. The solution was then centrifuged at 16,000 × g for 10 min, and the pellet was resuspended in 100 mM ammonium bicarbonate buffer. Subsequently, trypsin digestion was performed at an enzyme-to-protein ratio of 1:50 (w/w) overnight at 37°C. The digestion was quenched with 1% trifluoroacetic acid (TFA) (v/v), and the mixture was centrifuged at 16,000 × g at 4°C for 10 min. Concentration of the purified peptide was measured using a quantitative, colorimetric peptide assay kit (Thermo Fisher Scientific).

### Trypsin-catalyzed ^16^O/^18^O labeling

For enzymatic ^18^O-labeling, trypsin-digested peptides from the two groups were suspended in H_2_^18^O (97%, Cambridge Isotope Laboratories, USA), whereas peptides from the control group were suspended in H₂¹⁶O containing 20% ammonium acetate and trypsin (1:50, w/w). The samples were incubated for 24 h at 37°C. After labeling, the efficiency of ^18^O-labeling was measured using LC-MS/MS and analyzed using the MASCOT search engine (https://www.matrixscience.com/). Labeling efficiency was calculated as the ratio of labeled (^18^O) peptides to unlabeled peptides. The combined samples achieved > 98% labeling efficiency. The corresponding ¹⁶O (L) and ¹⁸O (H) peptides were then combined and centrifuged at 15,000 × g at 4°C. The supernatants were transferred to new tubes and subsequently lyophilized.

### Peptide fractionation by high-pH reverse-phase (RP) for global proteomics

RP fractionation was performed to reduce sample complexity and enable deep proteomic sequencing prior to liquid chromatography-mass spectrometry (LC/MS) analysis. A 1:1 mixture of ¹⁸O- and ¹⁶O-labeled peptides (100 µg) was fractionated using a high-pH reversed-phase peptide fractionation kit (Thermo Fisher Scientific). Briefly, 100 µL of peptides in 0.1% TFA were separated using a 5-50% acetonitrile (ACN) gradient in 0.1% triethylamine. The fractions were then centrifuged at 3,000 × g for 2 min to elute the peptides as flow-through fractions. The eluted peptides were desalted using C^18^ ZipTips (Millipore, Billerica, MA) based on the instructions of the manufacturer.

### Kac peptide enrichments and quantification

Peptides containing acetylated lysine (Kac) were sequentially enriched using an anti-acetyllysine antibody (PTM Biolabs, Inc., Chicago, IL). Briefly, 1 mg of dried peptides was dissolved in 500 μL of NETN buffer (100 mM NaCl, 1 mM EDTA, 50 mM Tris-HCl, 0.5% NP-40, pH 8.0), and insoluble particles were removed by centrifugation at 10,000 × g for 5 min at 4°C. The supernatant was incubated with 20 μL of Kac motif antibody at RT overnight with gentle rotation. After incubation, the beads were collected by centrifugation at 2,000 × g for 1 min. Subsequently, the beads were washed twice with NETN buffer and twice with ddH₂O. Peptides were eluted from the beads by incubation with 0.15% TFA for 10 min with gentle mixing. The supernatant was collected by centrifugation at 2,000 g for 1 min. The eluted samples were desalted using C_18_ columns, dried, and analyzed using LC-MS/MS.

### Nano-LC/MS Analysis

For global quantitative proteomics and Kac enrichment analyses, peptides were analyzed using two LC/MS devices: the LTQ Velos-Orbitrap for Kac samples and QExactive Plus Hybrid Quadrupole-Orbitrap for global proteomics. For the LTQ Velos-Orbitrap with a nanoflow LC system, Kac-enriched peptides were automatically loaded onto a homemade C₁₂ reversed-phase analytical column (75 μm × 120 mm, Jupiter C₁₂ resin, 90 Å pore size, 4 μm particle size; Phenomenex, Torrance, CA) by the autosampler nano-LC system. Peptides for global quantitative proteomics were directly loaded onto an Acclaim PepMap 100 C₁₈ HPLC column (75 μm × 2 cm, 3 µm nanoviper) as the loading column and an EASY-Spray PepMap RSLC C₁₈ column (75 μm × 50 cm, 2 µm; Thermo Fisher Scientific). Peptide separation was performed on the Ultimate 3000 RSLCnano system using a linear LC gradient of 5-28% solvent B (ACN/0.1% formic acid) for 0-110 min, 28-90% solvent B for 8 min, and 90% solvent B for 12 min at a flow rate of 300 nL/min. The eluted peptides were ionized and introduced into a Q Exactive Orbitrap spectrometer using a nanospray ionization source. For the LTQ Orbitrap, intact peptides with m/z values of 300‒1800 were analyzed at 1.8 kV, whereas peptides with m/z values of 350‒2000 were analyzed on the QE Plus Orbitrap at 2.0 kV. The ten most intense ions were sequentially isolated in MS1 and MS2 acquisition modes and subjected to high-energy collision dissociation with a normalized energy of 30%. Data-dependent scan parameters included an exclusion duration of 30s, a repeat count of two, and an exclusion window of +2 Da and -1 Da. Raw files from the datasets were uploaded to ProteomeX via the PRIDE partner repository under the identifier PXD047928 (https://www.ebi.ac.uk/pride).

### Bioinformatics

Gene Ontology (GO) term enrichment analysis was performed using the DAVID database (https://davidbioinformatics.nih.gov/). The following terms were analyzed with respect to the mouse proteome: Biological processes, molecular functions, and cellular compartments. Protein pathways were annotated using the Kyoto Encyclopedia of Genes and Genomes (KEGG) database. Differentially expressed proteins (DEPs) for global proteomics were identified using thresholds of < 0.5 and > 2. Motifs were extracted using pLOGO v1.2.0 (https://plogo.uconn.edu/). The global acetylation motif was generated using a 15-amino acid sequence window centered on the target lysine residue, with six neighboring amino acids on each side selected as the positive set. STRING networks were generated using the STRING database (https://string-db.org/) and clustered into seven groups based on the K-means algorithm to visualize the network.

### Statistical analysis

Statistical significance was assessed using ANOVA to compare both within- and between-group means. The results are expressed as mean ± standard error (SE).

## Supplementary Material

Supplementary figures and table.

## Figures and Tables

**Figure 1:**
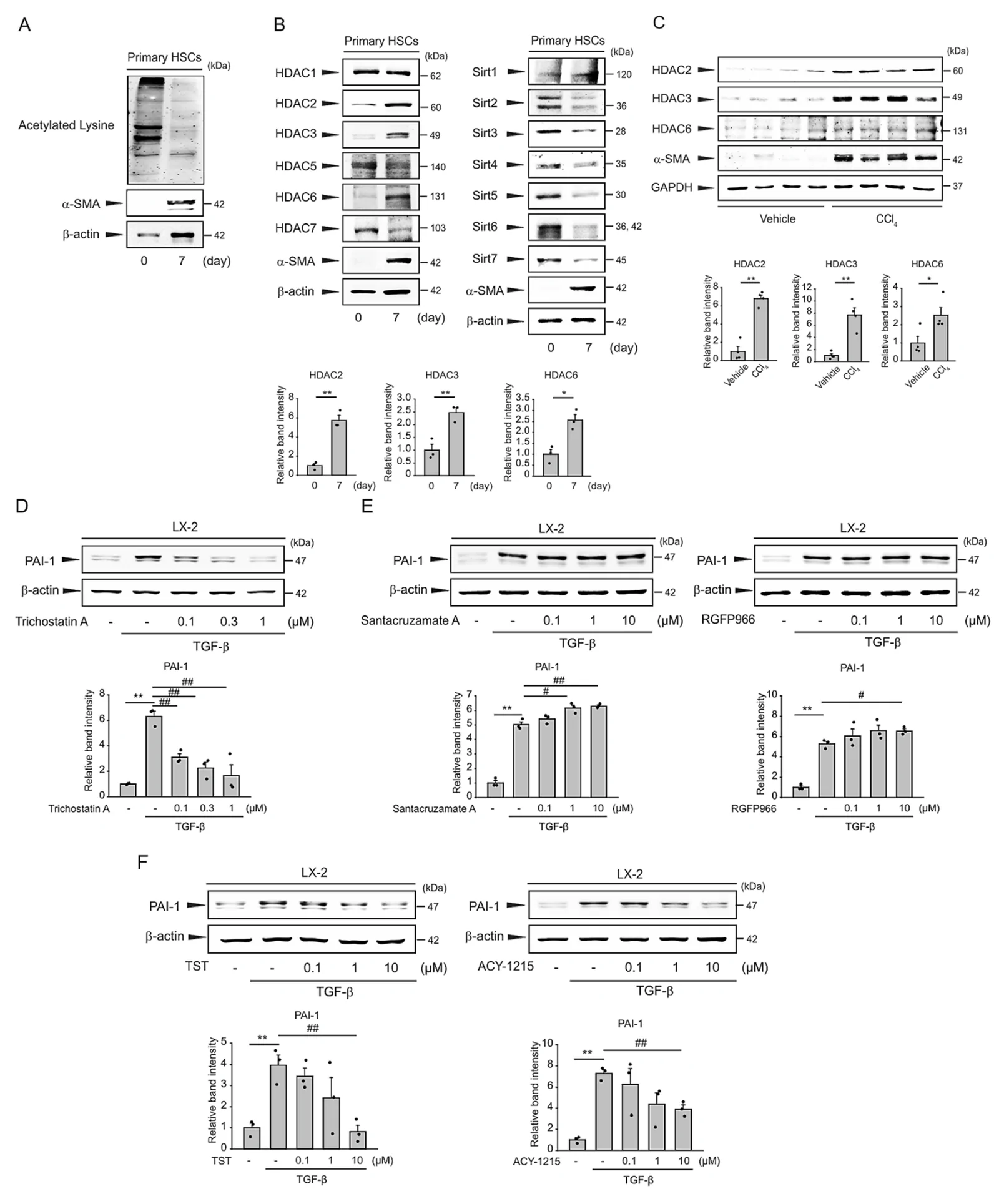
** HDACs associated with changes in acetylation level during HSC activation.** (A) Expression of acetylated lysine in quiescent and activated primary HSCs. Primary HSCs were isolated and cultured for 0 (quiescent HSCs) or 7 (activated HSCs) days. Acetylated lysine and α-SMA expression in the cell lysates were evaluated by immunoblotting. (B) Expression of HDACs and SIRTs in quiescent and activated primary HSCs. HDACs and SIRTs expression in the cell lysate was evaluated by immunoblotting. HDAC2, HDAC3, and HDAC6 levels were quantified using scanning densitometry. Data are presented as mean ± S.E. (*n* = 3; significant differences compared to quiescent HSCs, *p<0.05, **p<0.01). (C) Expression of HDAC2, HDAC3, and HDAC6 in liver tissues from CCl_4_-treated mice. HDAC2, HDAC3, and HDAC6 levels were quantified using scanning densitometry. Data are presented as the mean ± S.E. (*n* = 4; significant differences compared to vehicle, *p<0.05, **p<0.01). (D) Effect of the pan-HDAC inhibitor trichostatin A on fibrogenic gene expression. Cells were treated with trichostatin A (0.1, 0.3, 1 μM) in the presence or absence of TGF-β (1 ng/mL) for 6 h, and PAI-1 expression in the cell lysate was evaluated by immunoblotting. (E) Effect of santacruzamate A (HDAC2 selective inhibitor), and RGFP966 (HDAC3 inhibitor) on fibrogenic gene. Cells were treated with santacruzamate A (0.1‒10 μM) or RGFP966 (0.1‒10 μM) in the presence or absence of TGF-β (1 ng/mL) for 6 h, and PAI-1 expression in the cell lysate was evaluated by immunoblotting. (F) Effect of HDAC6 inhibitors on fibrogenic gene expression. Cells were treated with TST (0.1‒10 μM) or ACY-1215 (0.1‒10 μM) in the presence or absence of TGF-β (1 ng/mL) for 6 h; subsequently, PAI-1 expression in the cell lysate was evaluated by immunoblotting. (D‒F), PAI-1 level was quantified using scanning densitometry. Data are presented as mean ± S.E. (*n* = 3; significant differences compared to control, *p<0.05, **p<0.01; significant differences compared to TGF-β-treated cells, ^#^*p*<0.05, ^##^*p*<0.01).

**Figure 2:**
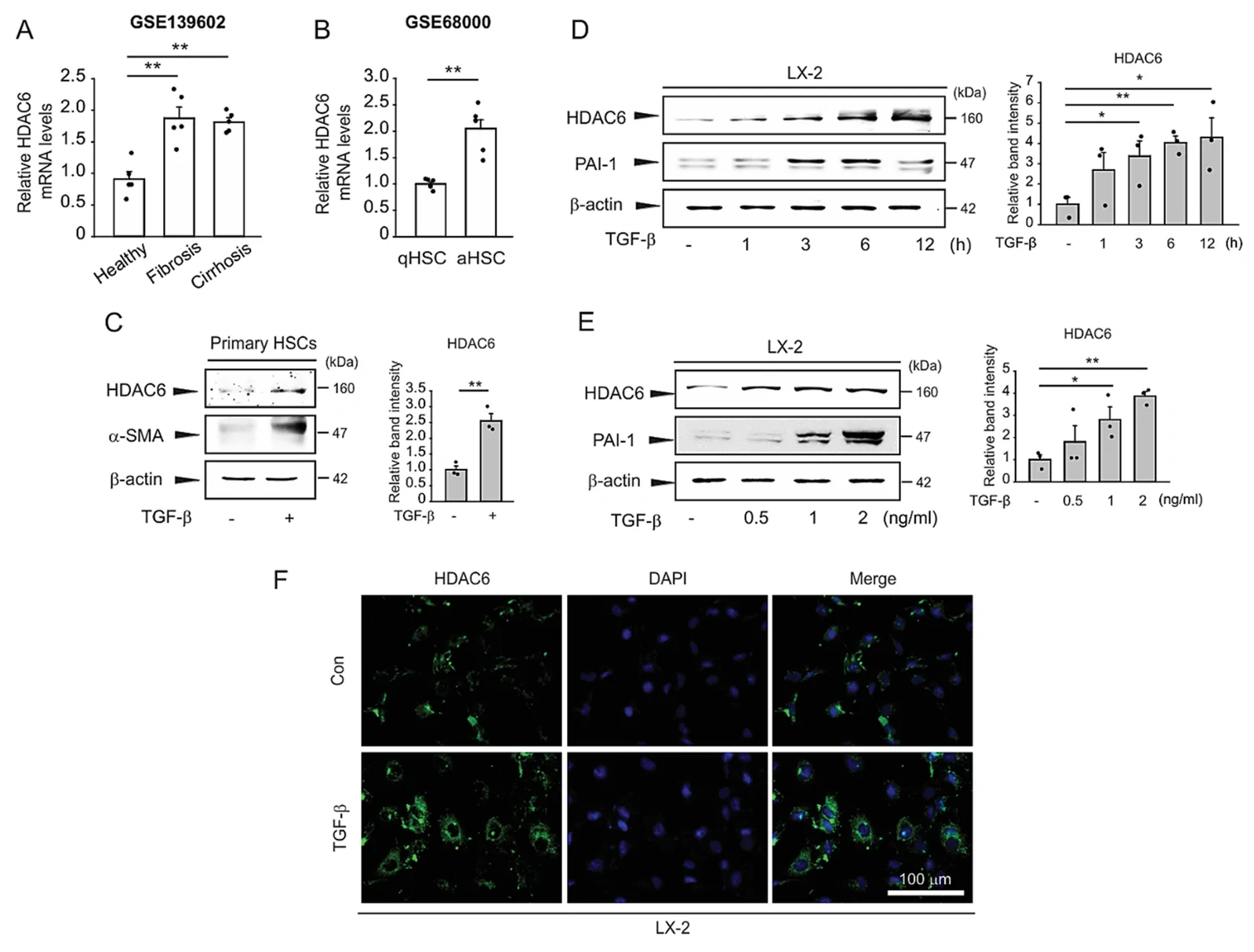
** HDAC6-mediated HSC activation.** (A) Transcript level of HDAC6 in patients at different liver disease stages from GSE139602. Data are expressed as mean ± SE (*n* = 5‒8; significant differences compared to Healthy, **p<0.01). (B) Transcript level of HDAC6 in quiescent or activated primary HSCs from GSE68000. Data are expressed as mean ± SE (*n* = 6; significant differences compared to quiescent HSCs, **p<0.01). (C) Effect of TGF-β on the expression of HDAC6 in primary HSCs. Primary HSCs were isolated from mice and treated with TGF-β (2 ng/mL) for 6 h, and HDAC6, α-SMA expression in the cell lysate was evaluated by immunoblotting. (D) Effect of TGF-β on HDAC6 expression. Cells were treated with TGF- β (2 ng/mL) for 1‒12 h; subsequently, HDAC6, PAI-1 expression in the cell lysate was evaluated by immunoblotting. (E) Effect of various concentrations of TGF- β on HDAC6 expression. Cells were treated with 0.5, 1, or 2 ng/mL TGF- β for 12 h, then HDAC6, PAI-1 expression in the cell lysate was evaluated by immunoblotting. (C‒E) HDAC6 level was quantified using scanning densitometry. Data are presented as the mean ± S.E. (*n* = 3; significant differences compared to control, *p<0.05, **p<0.01). (F) Immunostaining of HDAC6 in TGF-β-treated LX-2 cells (Scale bar=100 μm). Blue: DAPI, green: HDAC6.

**Figure 3:**
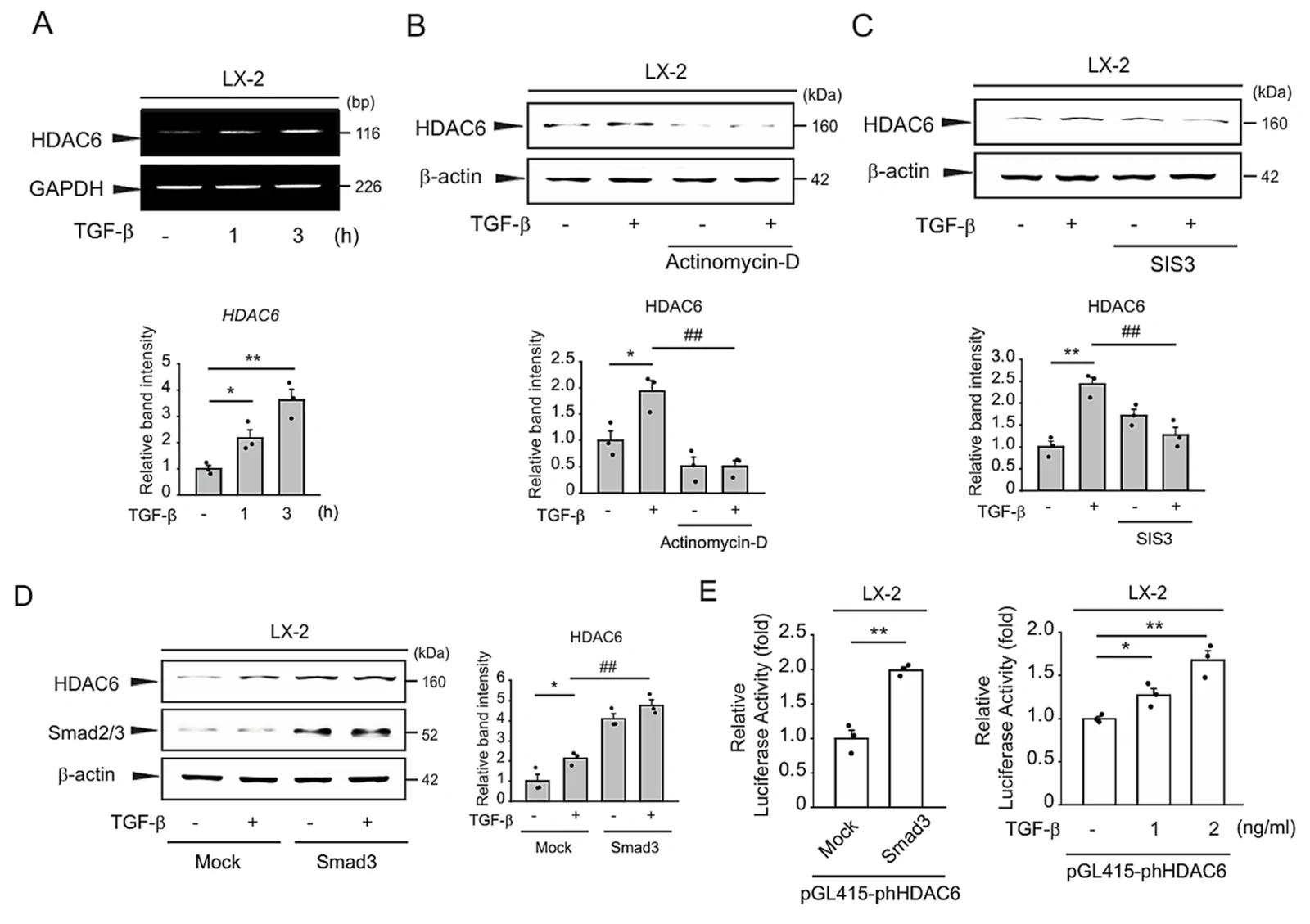
** Smad3-dependent HDAC6 induction by TGF-β.** (A) RT-PCR analysis. Cells were treated with TGF-β (2 ng/mL) for 1 or 3 h. Subsequently, HDAC6 mRNA levels were evaluated by RT-PCR. HDAC6 mRNA levels were quantified by scanning densitometry. (B) Effect of actinomycin-D on HDAC6 upregulation by TGF-β. Cells were treated with actinomycin-D (5 μg/mL) in the presence or absence of TGF-β (1 ng/mL) for 6 h, and HDAC6 expression in the cell lysate was evaluated by immunoblotting. HDAC6 level was assessed using scanning densitometry. (C) Effect of SIS3 on HDAC6 upregulation by TGF-β. Cells were treated with SIS3 (10 μM) in the presence or absence of TGF-β (1 ng/mL) for 6 h, then HDAC6 expression in the cell lysate was evaluated by immunoblotting. (A‒C), HDAC6 level was quantified using scanning densitometry. Data presented as the mean ± S.E. (*n* = 3; significant differences compared to control, *p<0.05, **p<0.01; significant differences compared to TGF-β-treated cells, ^##^*p*<0.01). (D) Effect of overexpression of Smad3 on HDAC6 upregulation by TGF-β. Cells were transfected with Mock or Smad3, and treated with TGF-β (1 ng/mL) for 6 h. HDAC6 and Smad2/3 expression in the cell lysate was evaluated by immunoblotting. HDAC6 level was quantified using scanning densitometry. Data are presented as mean ± S.E. (*n* = 3; significant differences compared to vehicle-transfected control cell, *p<0.05; significant differences compared to TGF-β-treated control cells, ^##^*p*<0.01). (E) HDAC6 promoter activity. Left: cells were transiently co-transfected with the phHDAC6 plasmid and Mock or Smad3. Right: cells were transfected with phHDAC6 plasmids and treated with TGF-β (1 or 2 ng/mL) for 12 h. Data are expressed as mean ± S.E. (*n* = 3; significant differences compared to vehicle-treated control cell, *p<0.05, **p<0.01).

**Figure 4:**
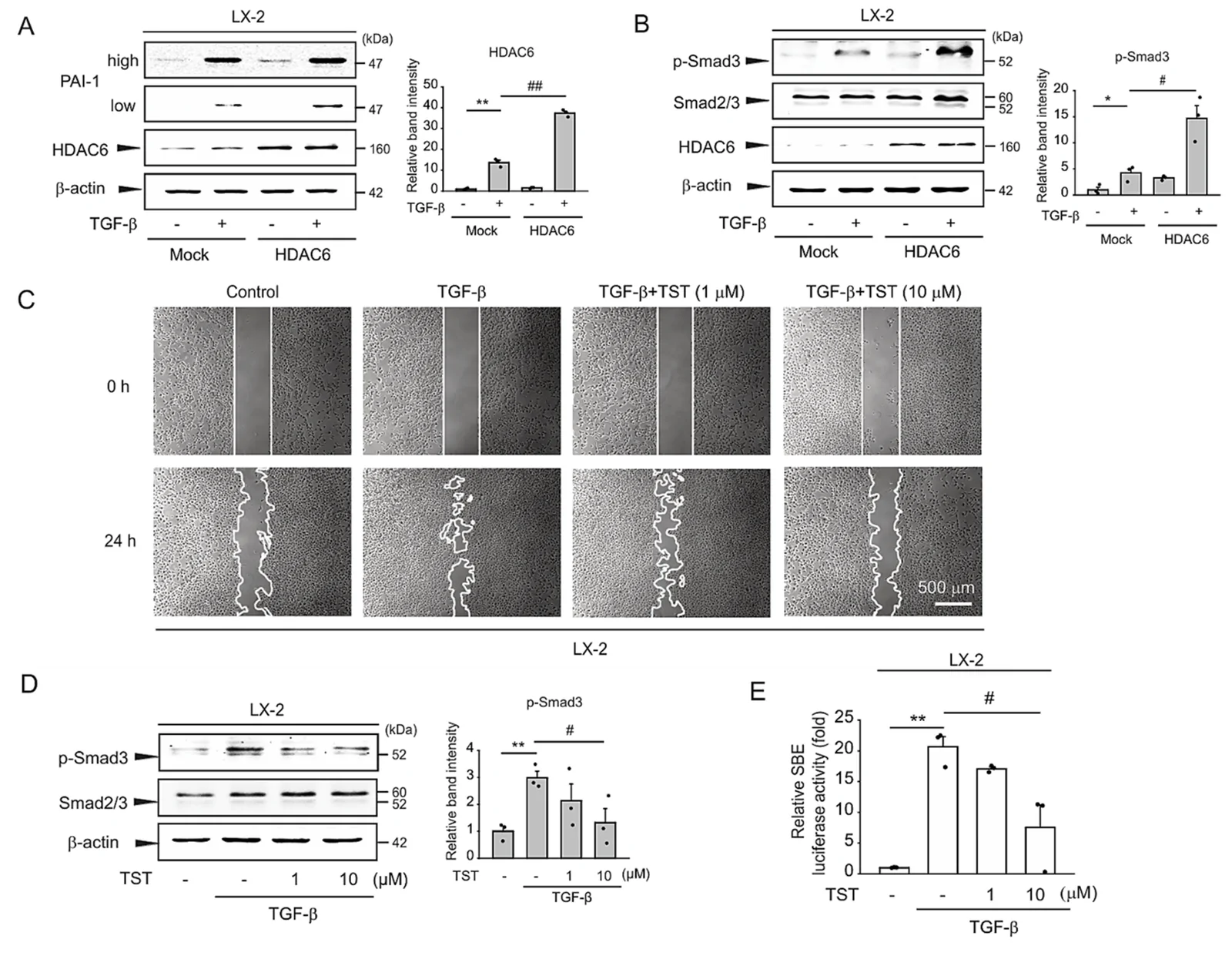
** Role of HDAC6 in HSC activation.** (A) Cells were transfected with Mock or HDAC6 and treated with TGF-β (1 ng/mL) for 6 h. PAI-1 and HDAC6 expression in the cell lysate was evaluated by immunoblotting. (B) Effect of HDAC6 overexpression on the activation of Smad3. Cells were transfected with Mock or HDAC6 and treated with TGF-β (1 ng/mL) for 30 min. Expression of p-Smad3, Smad2/3, and HDAC6 in the cell lysate was evaluated by immunoblotting. (A‒B) PAI-1 or p-Smad3 level was quantified using scanning densitometry. Data are presented as the mean ± S.E. (*n* = 3; significant differences compared to vehicle-treated control cells, *p<0.05, **p<0.01; significant differences compared to TGF-β-treated control cells, ^#^*p*<0.05, ^##^*p*<0.01, N.S., not significant). (C) Expression of TST on TGF-β-mediated cell migration based on the wound healing assay (Scale bar=500 μm). (D) Effect of TST on the activation of Smad3. Cells were treated with TST (10 μM) in the presence or absence of TGF-β (1 ng/mL) for 30 min, then p-Smad3, Smad2/3 expression in the cell lysate was evaluated by immunoblotting. p-Smad3 level was quantified using scanning densitometry. (E) Effect of TST on SBE luciferase activity. Cells were transfected with the SBE luciferase plasmid and treated with TST (10 μM) in the presence or absence of TGF-β (1 ng/mL) for 12 h. (D‒E) Data are presented as the mean ± S.E. (*n* = 3; significant differences compared to control, **p<0.01; significant differences compared to TGF-β-treated cells, ^#^*p*<0.05).

**Figure 5:**
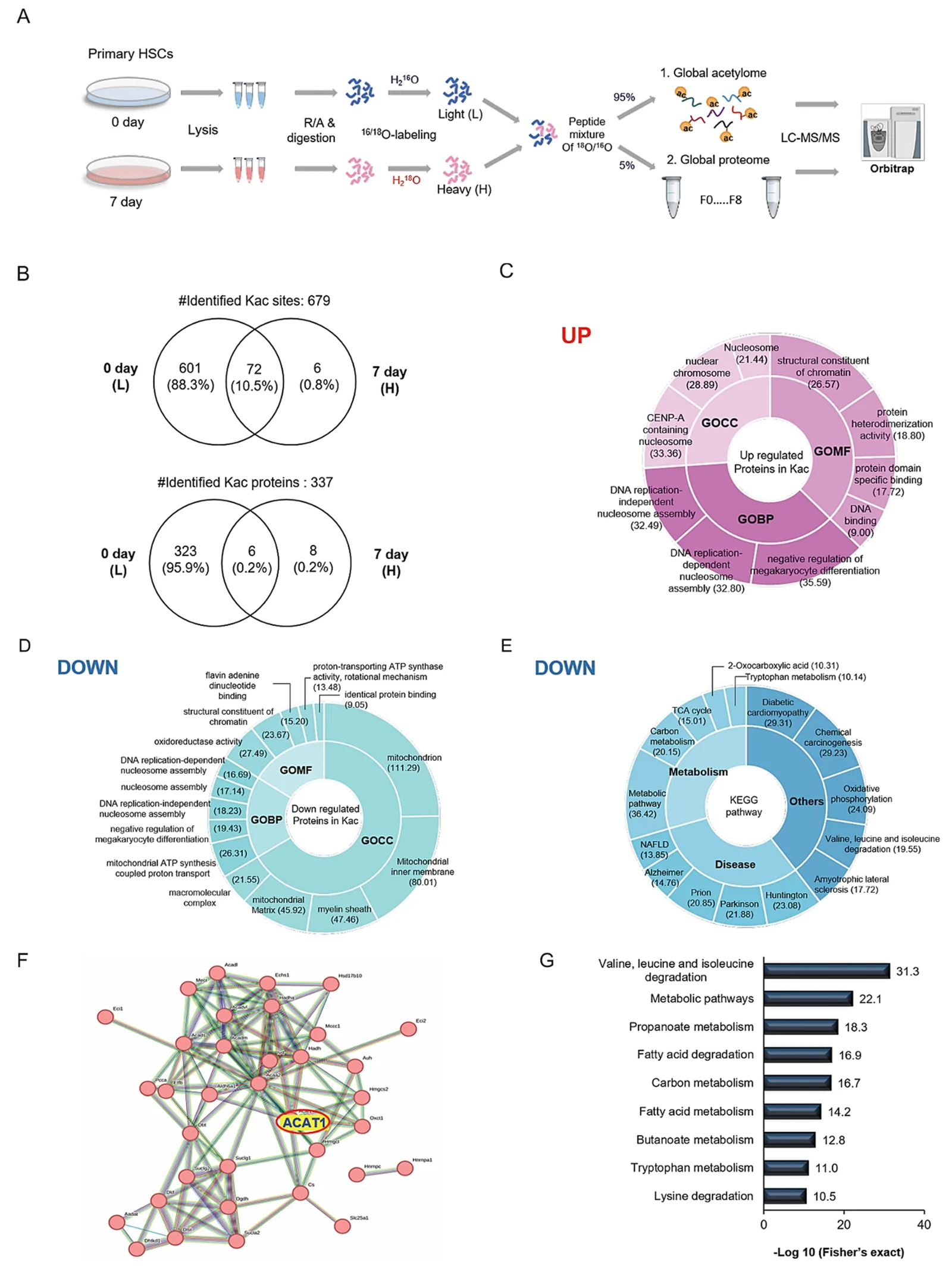
** Identification of HDAC6 substrates by global acetylome study.** (A) Schematic overview of experimental workflow for Kac enrichment and global proteomic analysis using ^16^O/^18^O enzymatic isotope labeling in the hepatic stellate cell (HSC) days 0 and 7 groups. (B) Venn diagram illustrating the overlap of the Kac protein site and protein number between HSC days 0 and 7. (C) Pie chart presenting Gene Ontology (GO) analysis of upregulated protein with Kac modification. (D) GO for downregulated proteins of Kac. (E) KEGG pathway of downregulated protein with Kac modification. (F) Interaction networks highlighting the regulatory role of acetyl-CoA acetyltransferase 1 (ACAT1) on Kac proteins. (G) KEGG pathway analysis specifically focusing on cluster 1.

**Figure 6:**
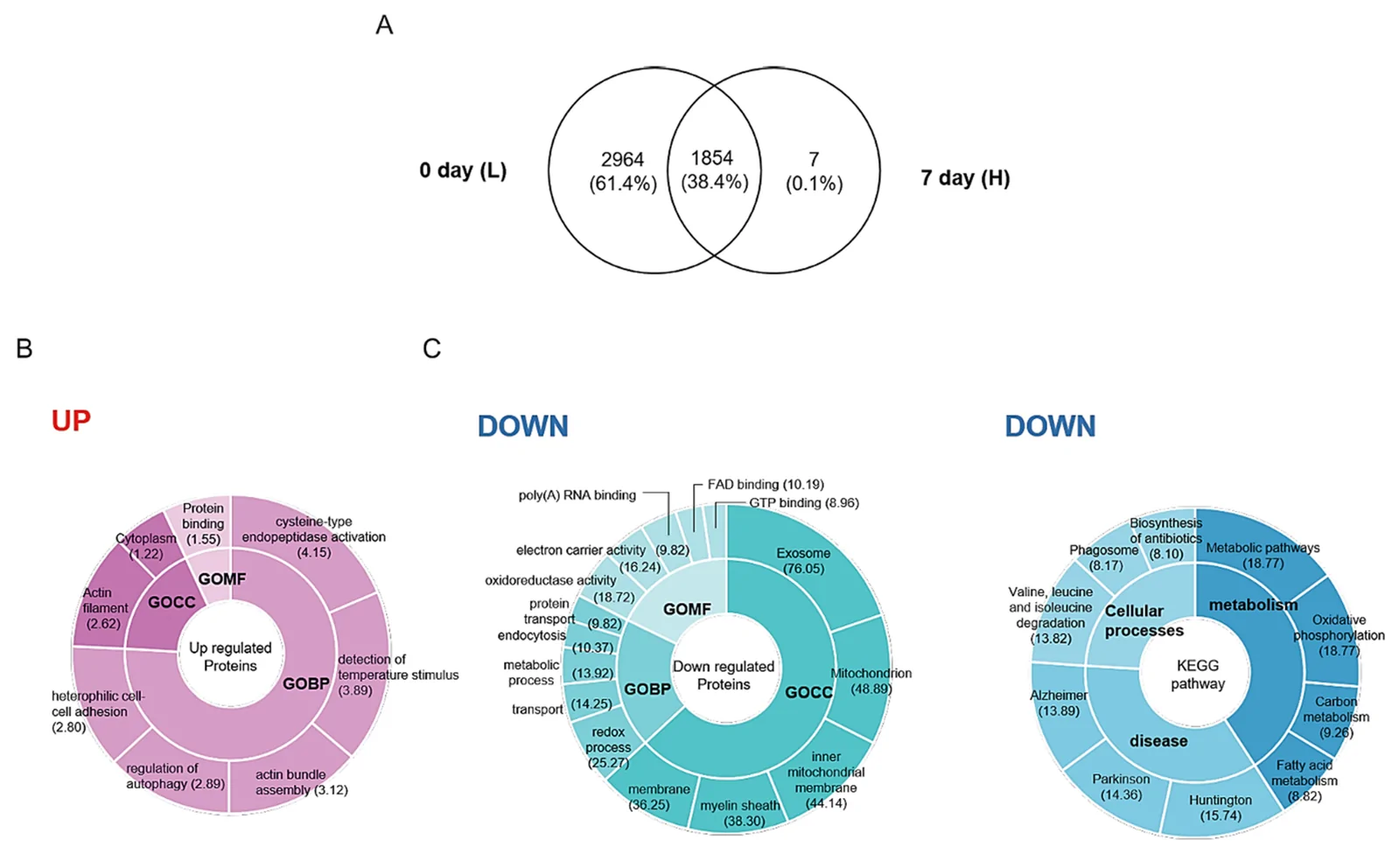
** GO and KEGG analyses of 446 differentially expressed proteins (DEPs) in global proteomics.** (A) Venn diagram illustrating the DEPs between HSC 0-day and 7-day groups. (B) Pie chart presenting Gene Ontology (GO) analysis resulting for upregulated protein. (C) GO and KEGG pathway analysis for downregulated proteins.

**Figure 7:**
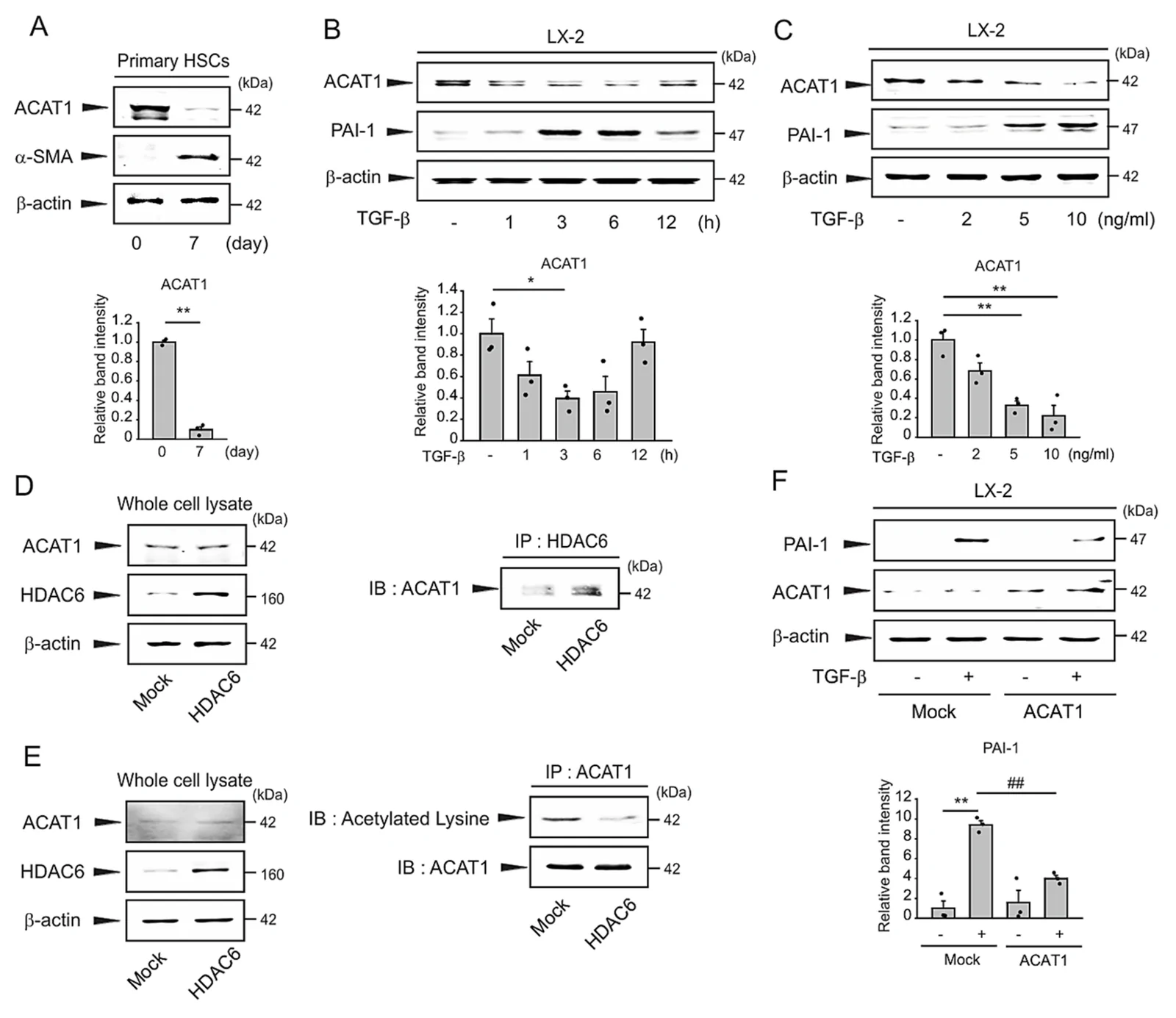
** Deacetylation of ACAT1 by HDAC6.** (A) Expression of ACAT1 in quiescent and activated primary HSCs. Primary HSCs were isolated and cultured for 0 (quiescent HSCs) or 7 (activated HSCs) days. ACAT1 and α-SMA expression in the cell lysate was evaluated by immunoblotting. ACAT1 level was quantified using scanning densitometry. Data are presented as the mean ± S.E. (*n* = 3; significant differences compared to quiescent HSCs, *p<0.05, **p<0.01). (B) Effect of TGF-β on ACAT1 expression. Cells were treated with TGF-β (2 ng/mL) for 1‒12 h, and ACAT1, PAI-1 expression in the cell lysate was evaluated by immunoblotting. (C) Effect of various concentrations of TGF-β on ACAT1 expression. Cells were treated with 0.5, 1, or 2 ng/mL TGF-β for 12 h, and ACAT1, PAI-1 expression in the cell lysate was evaluated by immunoblotting. (B‒C) ACAT1 level was quantified using scanning densitometry. Data are presented as mean ± S.E. (*n* = 3; significant differences compared to vehicle, *p<0.05, **p<0.01). (D) Immunoprecipitation. Cells were transfected with Mock or HDAC6. HDAC6 was immunoprecipitated with HDAC6 antibody and immunoblotted with ACAT1 antibody. (E) ACAT1 acetylation. Cells were transfected with Mock or HDAC6. ACAT1 was immunoprecipitated with ACAT1 antibody and immunoblotted with acetylated lysine antibody. (F) Effect of overexpression of ACAT1 on PAI-1 upregulation by TGF-β. PAI-1 level was quantified using scanning densitometry. Data are presented as mean ± S.E. (n = 3; significant differences compared to vehicle-treated control cells, **p<0.01; significant differences compared to TGF-β-treated control cells, ^##^p<0.01).

**Figure 8:**
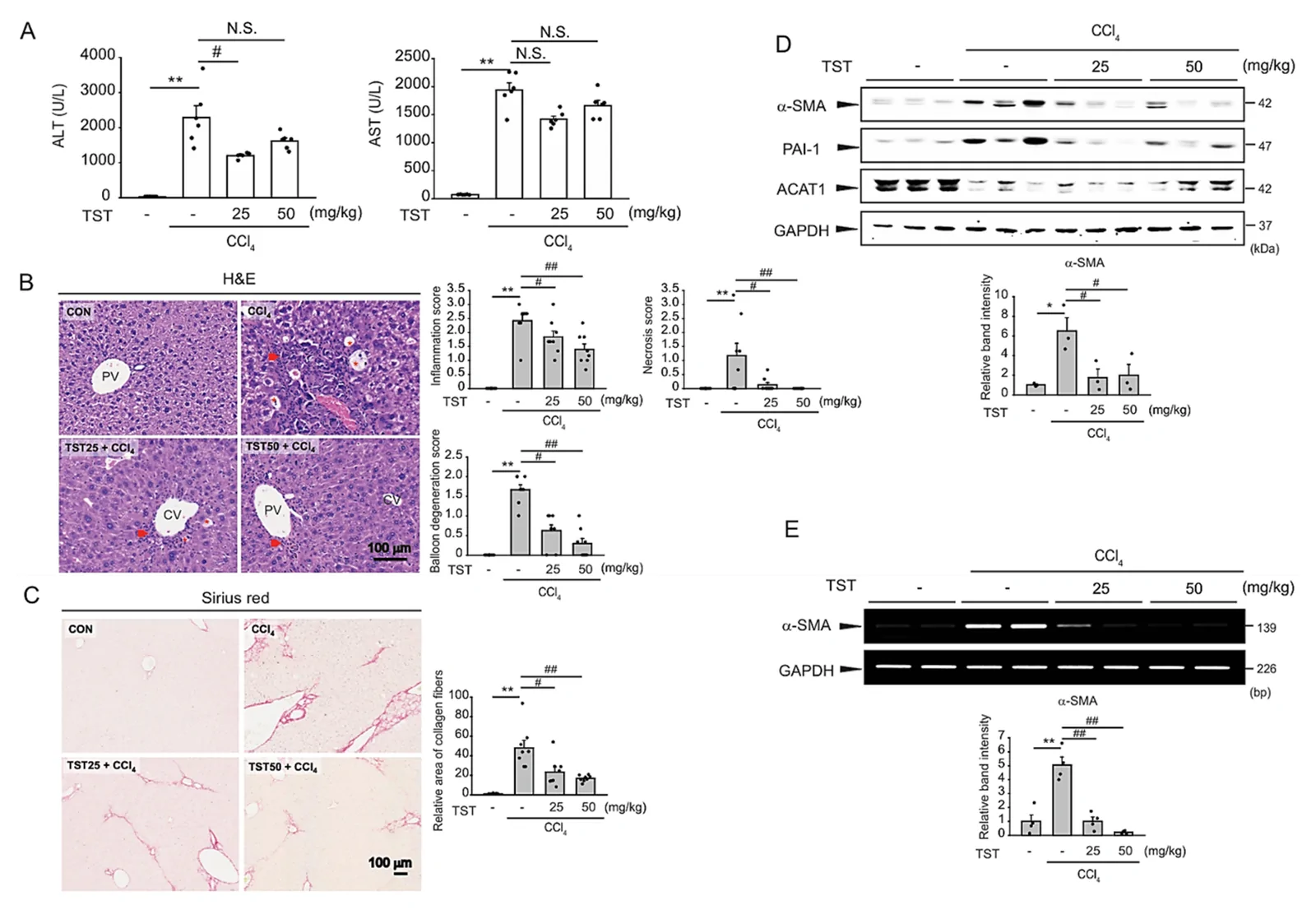
** Effect of TST on pathologic changes in a liver fibrosis animal model.** To induce liver fibrosis, CCl_4_ (0.5 mL/kg) was injected intraperitoneally into ICR mice three times per week for 2 weeks. Mice were orally administered TST (25 and 50 mg/kg) five times per week for the same period. (A) Serum ALT and AST levels. Data are presented as mean ± S.E. (*n* = 6; significant when compared to vehicle, **p<0.01; significant differences compared to CCl_4_, N.S., not significant). (B) Histopathological changes in mice with CCl_4_-induced liver injury, showing infiltration of inflammatory cells, including ballooning of hepatocytes and necrosis. CV, central vein; PV, portal vein; Red arrow, inflammatory cell infiltration; Red asterisk, hepatocyte vacuolation/ballooning cell, Scale bar=100 μm. (C) Representative images of Sirius red staining. Red: collagen fiber, Scale bar=100 μm. B and C data are presented as mean ± S.E. (*n* = 8 per group; significant differences compared to vehicle, *p<0.05, **p<0.01; significant differences compared to CCl_4_, ^#^*p*<0.05, ^##^*p*<0.01). (D) α-SMA and PAI-1, ACAT1 expression, evaluated by immunoblotting in liver homogenates of mice. These results represent the data for three mice in each group. α-SMA levels were quantified using scanning densitometry. Data are presented as mean ± S.E. (*n* = 3 per group; significant differences compared to vehicle, *p<0.05; significant differences compared to CCl_4_, ^#^*p*<0.05). (E) RT-PCR analysis for α-SMA in the liver homogenates of mice. The mRNA level of α-SMA was quantified using scanning densitometry. Data are presented as mean ± S.E. (*n* = 4 per group; significant differences compared to vehicle, *p<0.05, **p<0.01; significant differences compared to CCl_4_, ^##^*p*<0.01).

## Data Availability

Data are provided in the PRIDE database identifier PXD047928. Data can be accessed at https://www.ebi.ac.uk/pride/archive using the following reviewer credentials: Username, reviewer_pxd047928@ebi.ac.uk; Password, y4JKVGJL. Other data used and/or analyzed during the current study are available from the corresponding author on reasonable request.

## Abbreviations

ACAT1: Acetyl-CoA Acetyltransferase 1
ALT: Alanine aminotransferase
AP-1: Activator protein 1
AST: Aspartate aminotransferase
α-SMA: Alpha-smooth muscle actin
CCl_4_: Carbon tetrachloride
CHX: Cycloheximide
ECM: Extracellular matrix
HDAC: Histone deacetylase
HSCs: Hepatic stellate cells
H&E: Hematoxylin and eosin
PAI-1: Plasminogen activator inhibitor-1
PCR: Polymerase chain reaction
PTM: Post-translational modification
SBE: Smad-binding element
SIRT: Sirtuin
TGF-β: Transforming growth factor- β
TST: Tubastatin A
