# Supplementary material for: Histone acetyltransferases and deacetylase 6 (HDAC6) Promotes Hepatic Stellate Cell Activation and Liver Fibrosis
Source: Int J Biol Sci. 2026 Sep 10;22(15):8176–92. doi: 10.7150/ijbs.137447 (PMC13685615; doi:10.7150/ijbs.137447)
Supplement: Supplementary file 1 [file ijbsv22p8176s1.pdf]

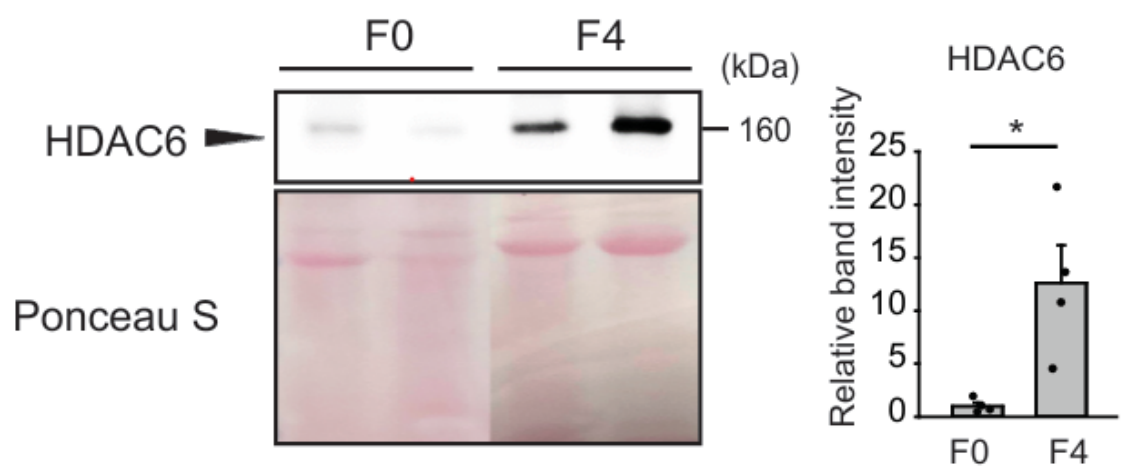

Figure S1. Immunoblot analysis. Expression of HDAC6 was evaluated by immunoblotting in liver homogenates of human. The data presented as the mean ± S.E. (n = 4, significant differences compared to F0, *p<0.05).

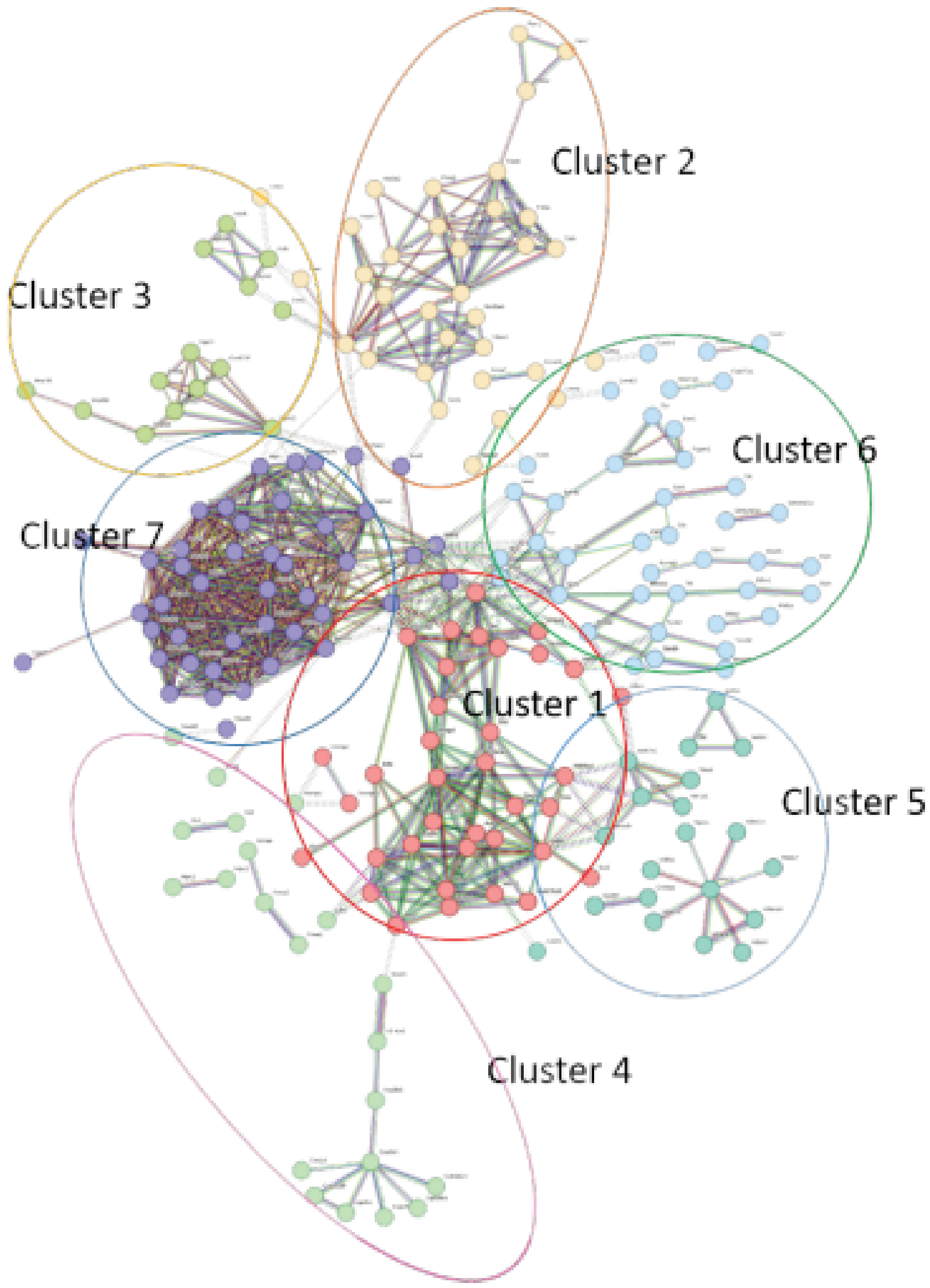

Figure S2. Seven clusters based on the K-means algorithm representing highly interconnected Kac protein networks using the STRING network.

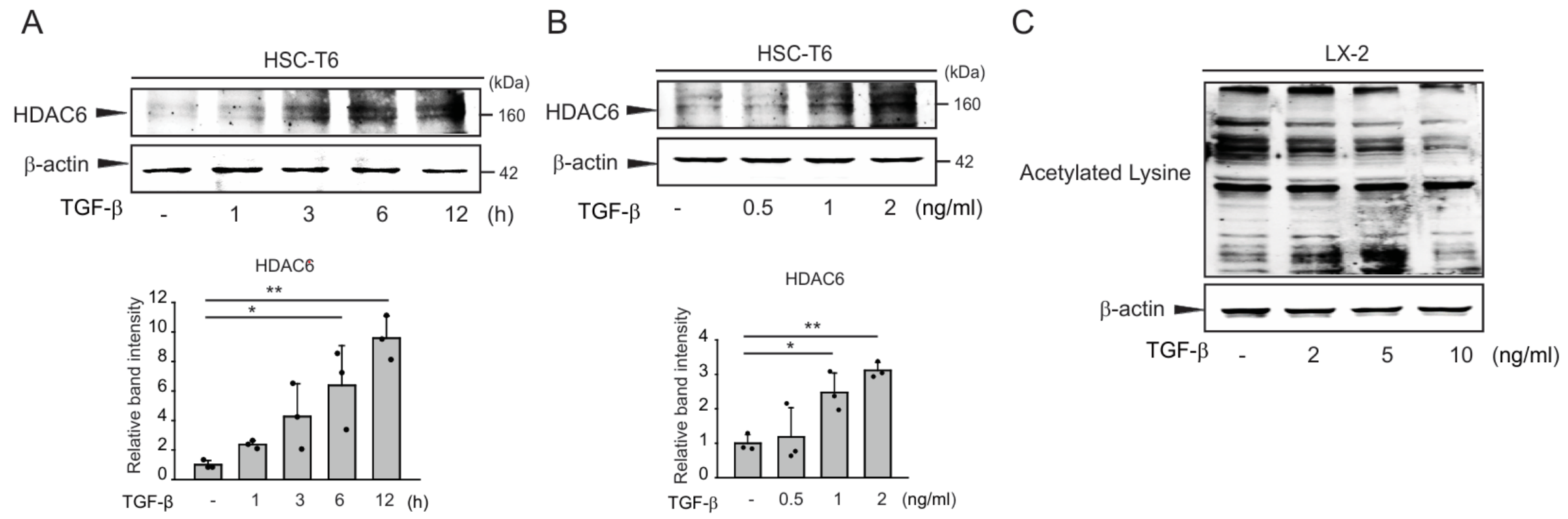

Figure S3. (A) Effect of TGF-β on the expression of HDAC6 in HSC-T6 cells. (B) Effect of a various concentrations of TGF- β on the HDAC6 expression in HSC-T6. For (A) and (B), HDAC6 level was quantified using scanning densitometry. The data presented as the mean ± S.E. (n = 3, significant differences compared to control, *p<0.05, **p<0.01). (C) Effect of TGF- β on the expression of acetylated lysine. Cells were treated with 2, 5, or 10 ng/mL TGF- β for 12 h, and then Acetylated lysine expression in the cell lysate was evaluated by immunoblotting.

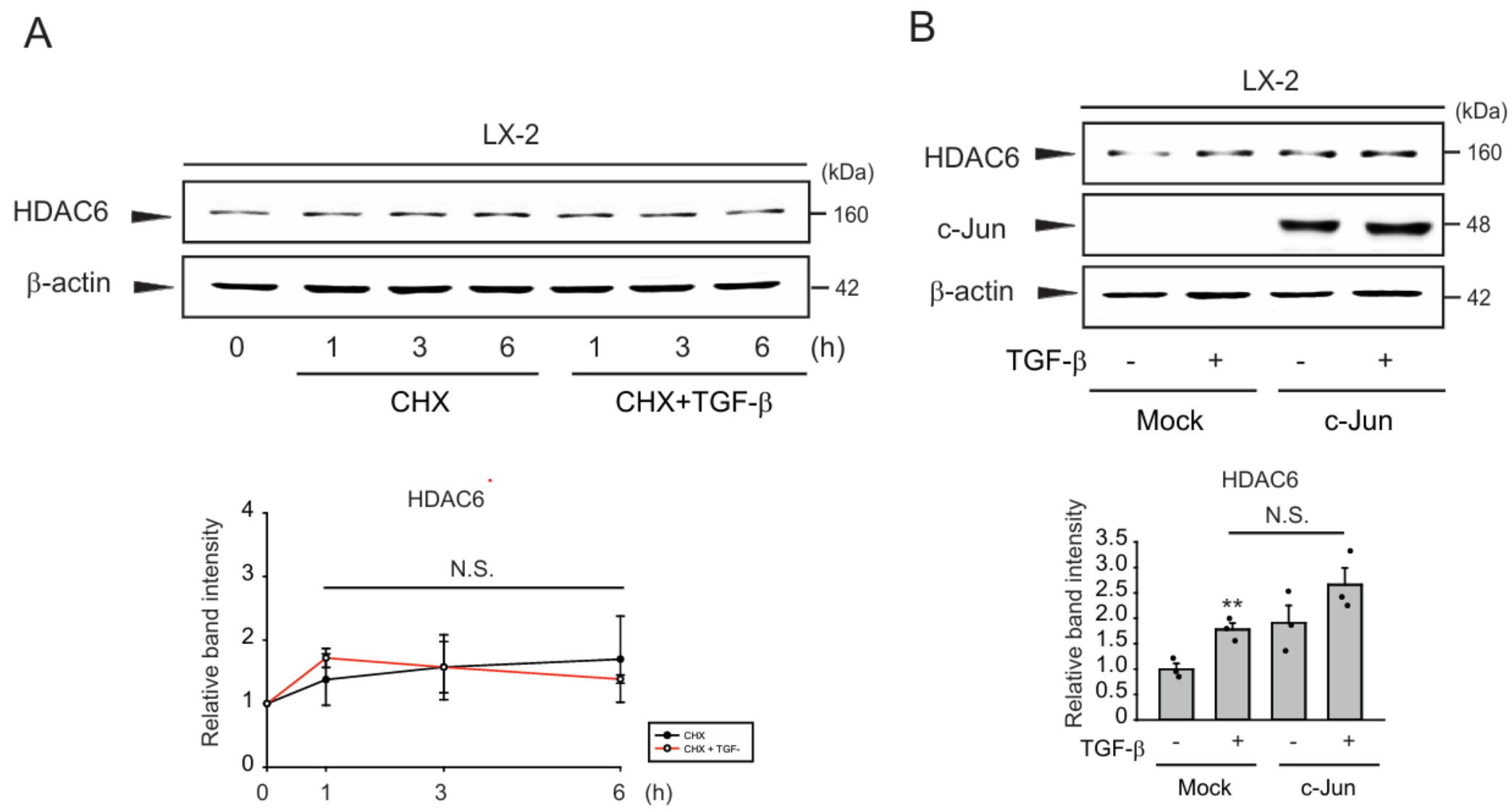

Figure S4. (A) Effect of CHX on TGF-β-mediated HDAC6 overexpression. Cells were treated with cycloheximide (CHX) (0.5 μg/mL) in the presence or absence of TGF-β (1 ng/mL) for the indicated periods. HDAC6 expression in the cell lysate was evaluated by immunoblotting. The data presented as the mean ± S.E. (*n* = 3, significant differences compared to control, N.S., not significant). (B) Effect of overexpression of c-Jun on HDAC6 upregulation by TGF-β. For (B), HDAC6 level was quantified using scanning densitometry. The data presented as the mean ± S.E. (*n* = 3, significant differences compared to vehicle transfected control cell, ***p*<0.01; significant differences compared to TGF-β treated control cells, N.S., not significant).

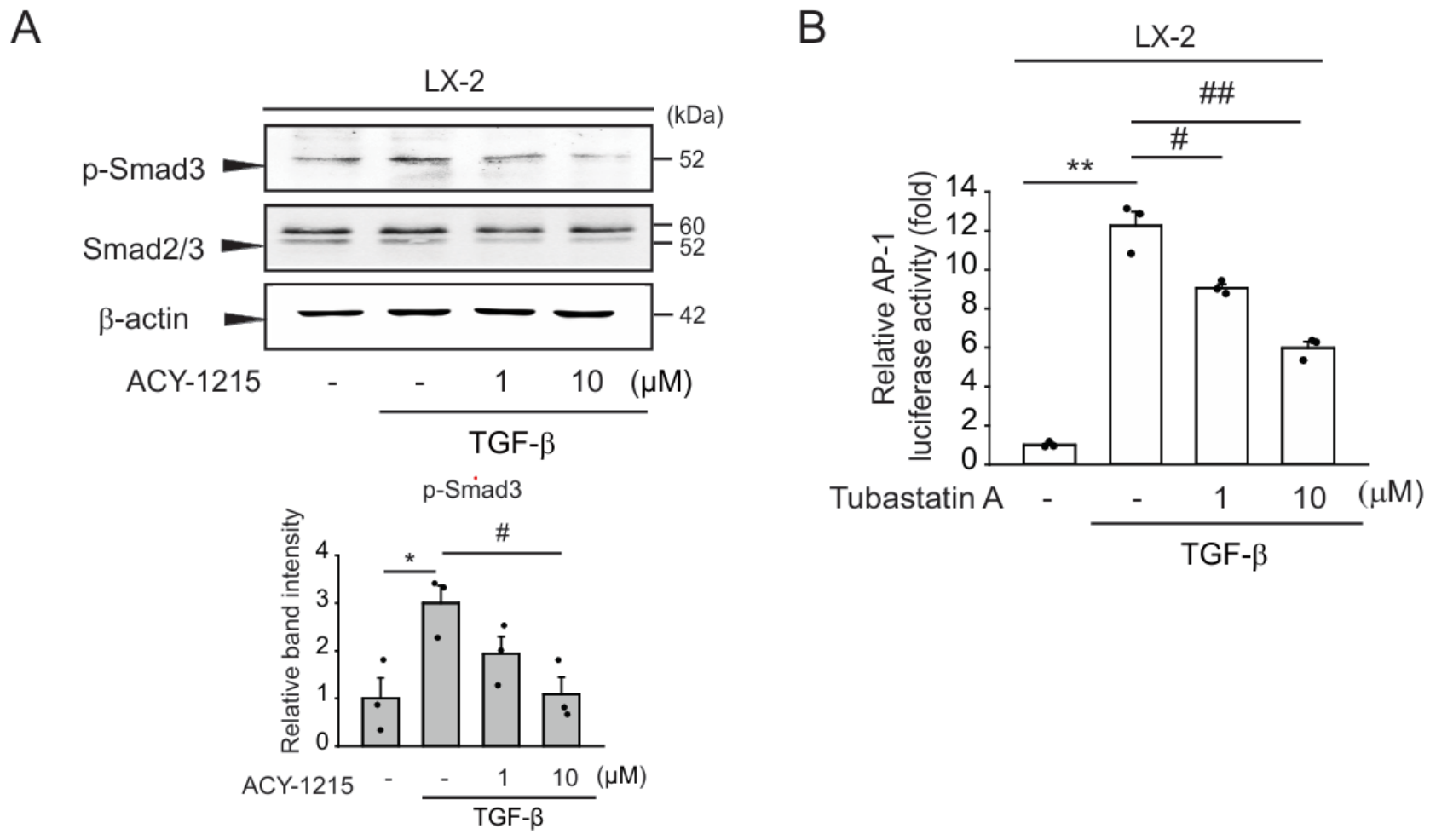

Figure S5. (A) Effect of ACY-1215 on the activation of Smad3. Cells were treated with ACY-1215 (10 μM) in the presence or absence of TGF-β (1 ng/mL) for 30 min, and then p-Smad3, Smad2/3 expression in the cell lysate was evaluated by immunoblotting. P-Smad3 level was quantified using scanning densitometry. (B) Effect of TST on AP-1 luciferase activity. For (A) and (B), data presented as the mean ± S.E. (n = 3, significant differences compared to control, *p<0.05, **p<0.01; significant differences compared to TGF-β treated cells, $^{\#}p$<0.05, $^{\#\#}p$<0.01).

Table S1. ACAT1 site information in Kac enriched proteins

**Proteins**	**Gene name**	**Positions within proteins**	**Localization prob**	**Amino acid**	**Sequences**	**Kac 7day/0day**
Q8QZT1	Acat1	242	1	K	G**Kac**PDVVVK	0.073
Q8QZT1	Acat1	187	1	K	DGLTDVYN**Kac**IHMGNCAENTAK	0.12
Q8QZT1	Acat1	260	1	K	VDFS**Kac**VPK	0.25
Q8QZT1	Acat1	220	1	K	S**Kac**EAWDAGK	Only 0 day
Q8QZT1	Acat1	227	1	K	EAWDAG**Kac**FASEITPITISVK	Only 0 day
Q8QZT1	Acat1	265	1	K	L**Kac**TVFQK	Only 0 day
Q8QZT1	Acat1	171	1	K	GATPYGGV**Kac**LEDLIVK	Only 0 day
